# Post-surgical inhibition of phosphatidylinositol 3-kinase attenuates the plantar incision-induced postoperative pain behavior via spinal Akt activation in male mice

**DOI:** 10.1186/s12868-019-0521-9

**Published:** 2019-07-31

**Authors:** Bing Xu, Cheng Mo, Chengmei Lv, Susu Liu, Jun Li, Jieying Chen, Yanhong Wei, Hongwei An, Li Ma, Xuehai Guan

**Affiliations:** 1 Department of Rehabilitation, The People’s Hospital of Guangxi Zhuang Autonomous Region, Nanning, 530021 Guangxi People’s Republic of China; 2 Department of Anesthesiology, The People’s Hospital of Guangxi Zhuang Autonomous Region, 6 Taoyuan Road, Nanning, 530021 Guangxi People’s Republic of China; 3 Department of Neurology, Liuzhou Traditional Chinese Medical Hospital, The Third Affiliated Hospital of Guangxi University of Chinese Medicine, Liuzhou, 545001 Guangxi People’s Republic of China; 4 The Centre of Pain Medicine, Guangxi Medical University, Nanning, 530007 Guangxi People’s Republic of China

**Keywords:** Postoperative pain, Plantar incision, Phosphatidylinositol 3-kinase, Akt, Spinal dorsal horn

## Abstract

**Background:**

Postoperative pain (POP) is a severe acute pain encountered in patients suffering from an operation, and is less than adequately controlled by the currently available analgesics. Phosphatidylinositol 3-kinase (PI3K) has been reported to have an important role in neuropathic and inflammatory pain. Our previous research revealed that pre-surgical inhibition of spinal PI3K alleviated the pain behavior induced by plantar incision in mice. The aim of this study was to clarify whether post-surgical inhibition of PI3K would attenuate the POP and the underlying mechanisms.

**Methods:**

A POP model was established by plantar incision in Kunming mice. A behavioral test was performed to determine mechanical allodynia, thermal hyperalgesia, and cumulative pain scores. The spinal Fos was detected by immunohistochemistry. The spinal expression of protein kinase B (Akt) or phosphorylated Akt (pAkt) was explored using western blot. The cellular location of pAkt was determined by immunofluorescence.

**Results:**

Post-surgical inhibition of PI3K attenuated mechanical allodynia, thermal hyperalgesia, and cumulative pain scores induced by plantar incision significantly in male mice, and mildly in female mice. Post-surgical inhibition of PI3K attenuated the expression of spinal Fos in male mice. Plantar incision induced a time-dependent expression of spinal pAkt in male mice, which was primarily expressed in the spinal dorsal horn, and localized with the neuron and microglia’s marker. Post-surgical inhibition of PI3K attenuated the activation of Akt induced by plantar incision in male mice as well.

**Conclusions:**

We concluded that post-surgical inhibition of PI3K could attenuate the pain-related behaviors induced by plantar incision, by suppressing the activation of spinal Akt in male mice. This finding might be used in clinical studies to reach a better understanding of POP mechanisms and optimal treatment.

## Background

With advanced surgical procedures, the survival of patients has improved recently. However, postoperative pain (POP) is still intractable, and greatly influences the quality of life, lengthens the hospital stay, increases morbidity and costs, and delays recovery. Opioids are used to alleviate pain; however, usage of higher doses of opioids is a limitation owing to the tolerability levels and side effects such as vomiting, nausea, ileus, sedation, and respiratory depression [1]. The implementation of enhanced recovery after surgery (ERAS) protocols is aimed at promoting opioid-free and multimodal analgesia, in the context of providing adequate pain relief [2–4]. Therefore, developing novel and potent non-opioid drugs for controlling severe POP, is much needed.

Phosphatidylinositol 3-kinase (PI3K) has been shown to play a key role in a plethora of pathological and physiological processes. Furthermore, it has recently been reported that PI3K and its downstream protein kinase B (Akt), expressed in dorsal root ganglion and spinal dorsal horn, are involved in the modulation of nociceptive information, such as neuropathic pain, inflammatory pain, and bone cancer pain. We have previously shown that pre-surgical inhibition of spinal PI3K alleviated the pain behavior induced by plantar incision in mice [5]. Post-operative analgesia is more critical than pre-operative analgesia; however, whether post-surgical inhibition of PI3K could attenuate the POP is unknown, and the plausible mechanisms involved remain elusive. To verify these possibilities, we explored the analgesic effect of post-surgical inhibition of PI3K and the underlying mechanism by combining behavioral and molecular biological methods.

## Results

### Post-surgical inhibition of PI3K significantly attenuated mechanical allodynia, thermal hyperalgesia, and cumulative pain scores induced by plantar incision in male mice

**Fig. 1:**
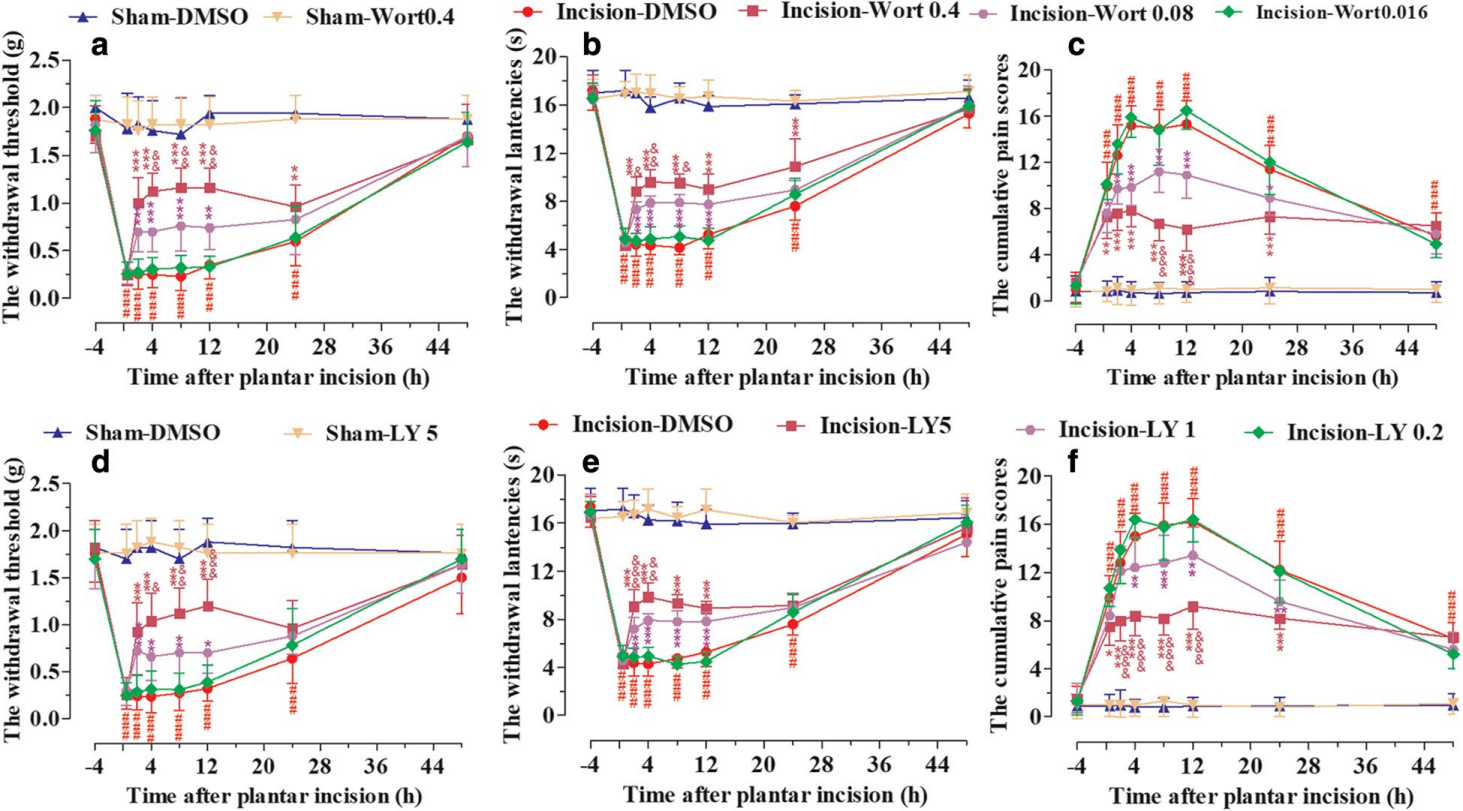
PI3K inhibitor attenuated mechanical allodynia, thermal hyperalgesia, and cumulative pain scores induced by plantar incision in male mice. Wortmannin (0.016, 0.08, and 0.4 μg), LY294002 (0.2, 1, and 5 μg) or DMSO was intrathecally injected at 90 min after plantar incision. Paw withdrawal threshold (PWT) to mechanical stimuli, paw withdrawal latency (PWL) to radiant heat, and cumulative pain scores (CPS) were recorded at 0.5, 2, 4, 8, 12, 24, and 48 h after plantar incision. Post-surgical inhibition of PI3K with various doses of wortmannin (**a**–**c**) or LY294002 (**d**–**f**) attenuated the reduction of PWT (**a**, **d**) and PWL (**b**, **e**), or the induction of CPS (**c**, **f**) induced by plantar incision in a dose-dependent manner. ^###^*P *< 0.001, compared with Sham-DMSO group; *P < 0.05, **P < 0.01, ***P < 0.001, compared with incision-DMSO group; ^&^*P *< 0.05, ^&&^*P *< 0.01, ^&&&^*P *< 0.001, compared with Incision-Wort 0.08 group or Incision-LY 1 group (two-way repeated measure ANOVA followed by Bonferroni’s post-test; *n *= 10 mice in each group). Data were presented as mean ± SEM. *Wort* wortmannin, *LY* LY294002

Plantar incision activated PI3K pathway, and pre-surgical treatment with PI3K inhibitor wortmannin or LY294002 prevented pain behavior induced by plantar incision [5]. In order to explore whether post-surgical inhibition of PI3K would attenuate the pain behavior in male mice, wortmannin (0.016, 0.08, and 0.4 μg/5 μl), LY294002 (0.2, 1, and 5 μg/5 μl), or DMSO (5%, 5 μl) was injected intrathecally at 90 min after plantar incision. Paw withdrawal threshold (PWT) to mechanical stimuli, paw withdrawal latency (PWL) to radiant heat, and accumulative pain score (CPS) were recorded at 0.5, 2, 4, 8, 12, 24, and 48 h after plantar incision. Post-surgical treatment with various doses of wortmannin (Fig. 1a–c) or LY294002 (Fig. 1d–f) attenuated the reduction of PWT (Fig. 1a, d) and PWL (Fig. 1b, e), or the induction of CPS (Fig. 1c, f) in a dose-dependent manner. Intrathecal vehicle treatment (5% DMSO) did not affect the pain-related behavior at the experimental time points.

### Post-surgical inhibition of PI3K mildly attenuated mechanical allodynia, thermal hyperalgesia, and cumulative pain scores induced by plantar incision in female mice

**Fig. 2:**
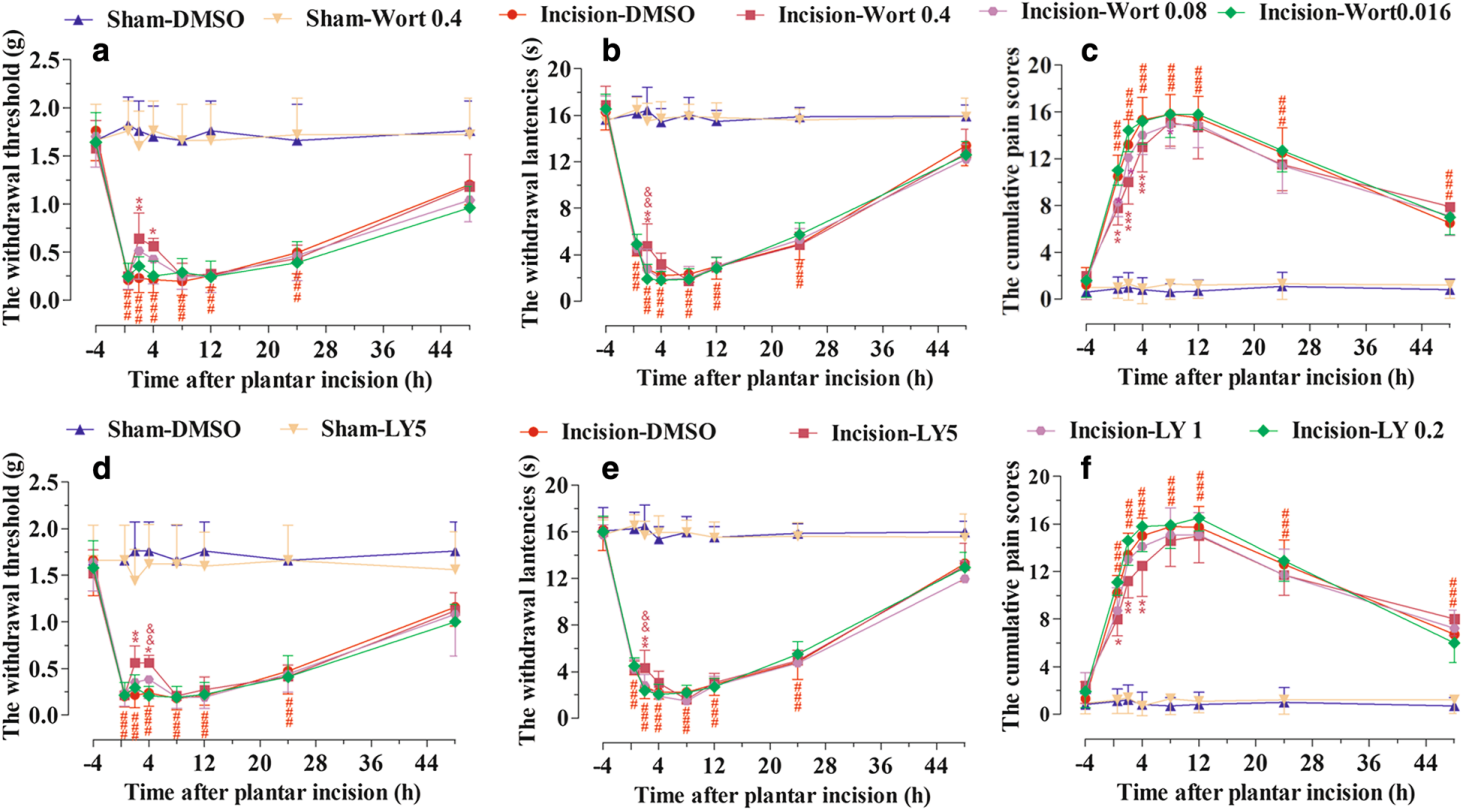
PI3K inhibitors mildly attenuated mechanical allodynia, thermal hyperalgesia, and cumulative pain scores induced by plantar incision in female mice. PI3K inhibitor wortmannin (0.016, 0.08, and 0.4 μg), LY294002 (0.2, 1, and 5 μg), or DMSO was intrathecally injected at 90 min after plantar incision. PWT to mechanical stimuli, PWL to radiant heat and CPS were recorded at 0.5, 2, 4, 8, 12, 24, and 48 h after plantar incision. Post-surgical inhibition of PI3K with various doses of wortmannin (**a**–**c**) or LY294002 (**d**–**f**) attenuated the reduction of PWT (**a**, **d**) and PWL (**b**, **e**), or the induction of CPS (**c**, **f**) induced by plantar incision mildly. ^###^*P *< 0.001, compared with Sham-DMSO group; *P < 0.05, **P < 0.01, ***P < 0.001, compared with Incision-DMSO group; ^&^*P *< 0.05, ^&&^*P *< 0.01, ^&&&^*P *< 0.001, compared with Incision-Wort 0.08 group or Incision-LY 1 group (two-way repeated measure ANOVA followed by Bonferroni’s post-test; *n *= 10 mice in each group). Data were presented as mean ± SEM. *Wort* wortmannin, *LY* LY294002

Sex differences have been identified in clinical pain conditions, and influences of these differences on pain and analgesia have become a topic of preclinical and clinical interest. In order to explore whether post-surgical inhibition of PI3K would attenuate the pain behavior in female mice, wortmannin (0.016, 0.08, and 0.4 μg/5 μl), LY294002 (0.2, 1, and 5 μg/5 μl), or DMSO (5%, 5 μl) was intrathecally injected at 90 min after plantar incision in female mice. PWT to mechanical stimuli, PWL to radiant heat and CPS were recorded at 0.5, 2, 4, 8, 12, 24, and 48 h after plantar incision. Post-surgical inhibition of PI3K with various doses of wortmannin (Fig. 2a–c) or LY294002 (Fig. 2d–f) attenuated the reduction of PWT (Fig. 2a, d) and PWL (Fig. 2b, e), or the induction of CPS (Fig. 2c, f), mildly. Intrathecal vehicle treatment (5% DMSO) did not affect the pain-related behavior at the experimental time points.

### Post-surgical inhibition of PI3K attenuated the expression of spinal Fos induced by plantar incision in male mice

**Fig. 3:**
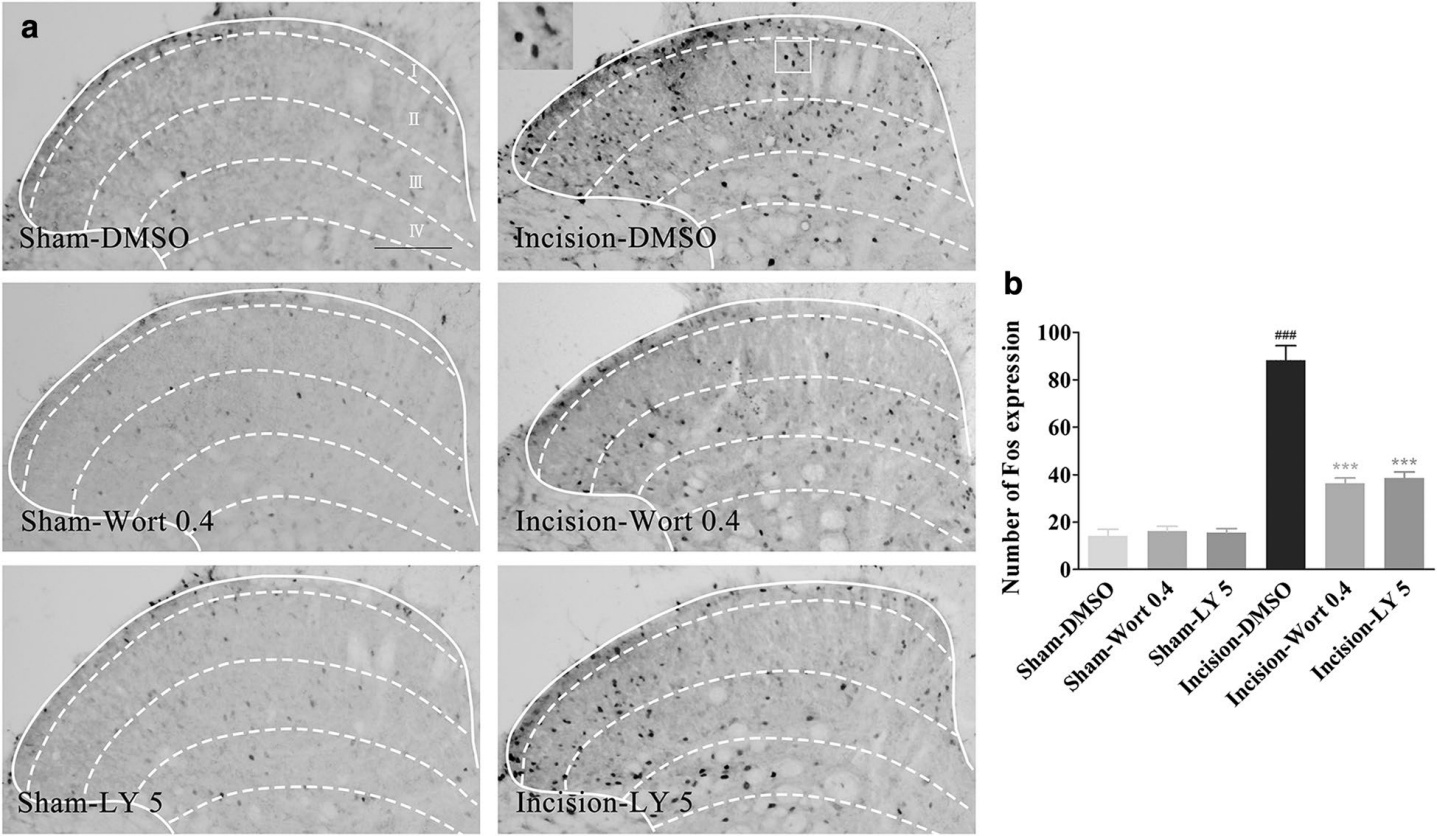
Post-surgical inhibition of PI3K attenuated the spinal Fos protein expression induced by plantar incision in male mice. PI3K inhibitor wortmannin (0.4 μg) or LY294002 (5 μg) was intrathecally injected 45 min after plantar incision. Spinal Fos protein expression was assayed at 2 h after plantar incision. **a** Representative immunohistochemical staining of Fos expression in the spinal cord. **b** Quantitative data of Fos expression in the spinal cord. ^###^*P *< 0.001, compared with Sham-DMSO group; ***P < 0.001, compared with Incision-DMSO group (one-way ANOVA, followed by Bonferroni’s multiple comparison test; *n *= 6 mice in each group). Data are presented as mean ± SEM. Scale bar = 200 μm

To clarify whether the analgesic effect of PI3K inhibitors on pain behavior is induced by plantar incision, we assayed the expression of spinal Fos protein after treatment with PI3K inhibitors. PI3K inhibitor wortmannin (0.4 μg/5 μl), LY294002 (5 μg/μl), or vehicle (5% DMSO), was intrathecally injected 45 min after plantar incision in male mice. Spinal Fos protein expression was analyzed at 2 h after plantar incision using immunohistochemistry. Two hours after plantar incision in the right foot, the number of Fos protein-positive cells increased significantly in the ipsilateral dorsal horn (*P *< 0.01), compared with the sham operation group (Fig. 3a, b). Post-surgical inhibition of PI3K with wortmannin or LY294002 intrathecally, partially attenuated the spinal Fos expression induced by plantar incision (Fig. 3a, b), while DMSO and wortmannin or LY294002 had no effect on the Fos expression in the sham operation group (Fig. 3a, b).

### Plantar incision induced a time-dependent activation of Akt in the spinal dorsal horn in male mice but not in female mice

**Fig. 4:**
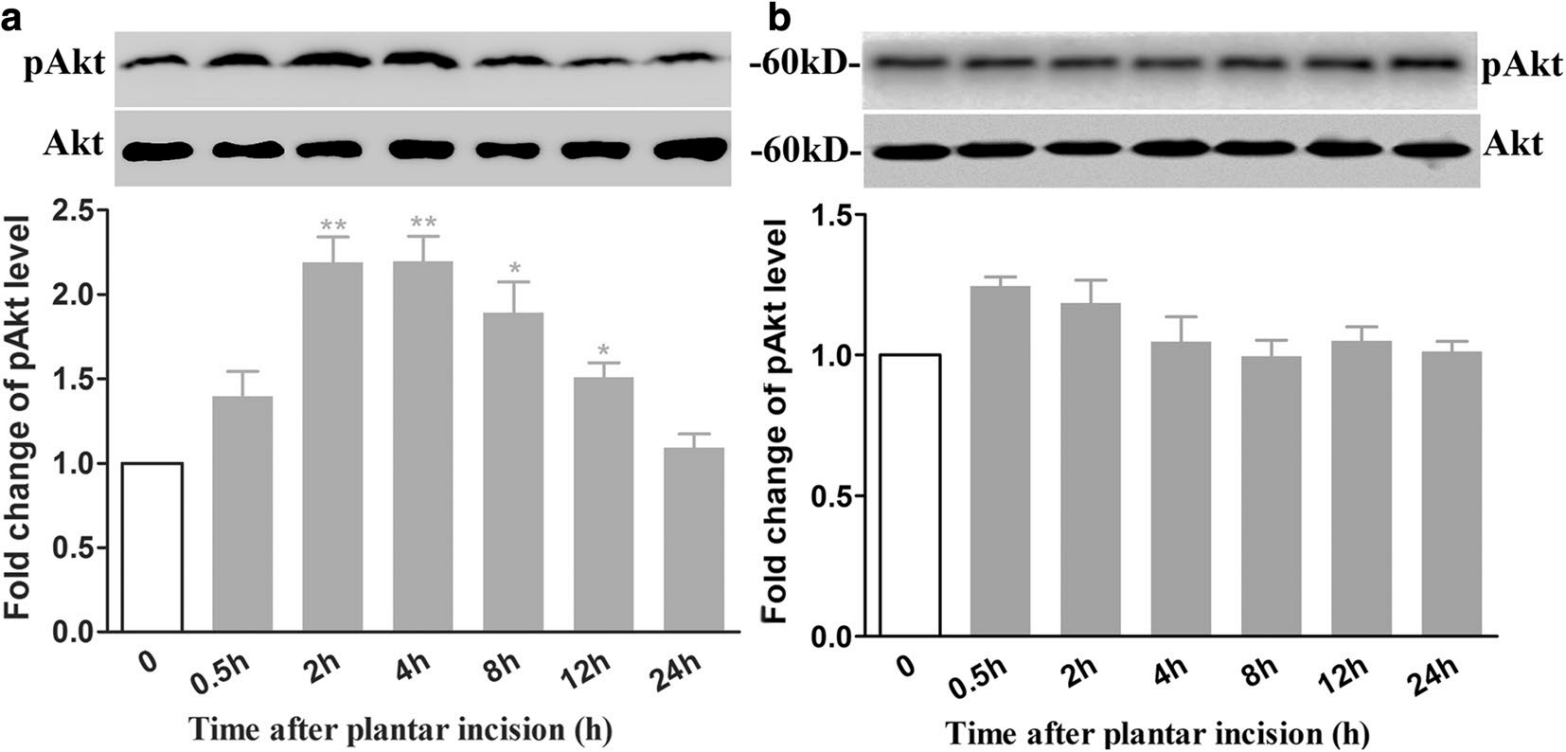
Plantar incision induced a time-dependent increase of spinal pAkt expression in male mice, but not in female mice. The expression of pAkt and Akt was assayed at 0, 0.5, 2, 4, 8, 12, 24 h time-points after plantar incision in male mice (**a**) or female mice (**b**). The representative bands (top) for the expression of pAkt in the spinal cord at different time points after plantar incision and the quantitative data (bottom) for the expression of pAkt are shown. The fold change for the density of pAkt was normalized to total Akt for each sample, respectively. The fold change of pAkt in the 0-time point group was set at 1 for quantification. *P < 0.05, **P < 0.01, compared with 0-time point group. (one-way ANOVA, followed by Bonferroni’s multiple comparison test; *n *= 4 mice in each group). Data are presented as mean ± SEM

To determine whether Akt pathway was activated by the plantar incision in mice, we assayed the time course of the expression of phosphorylated Akt (S473, pAkt) as the indicator of activation of Akt pathway in the spinal cord. The expression of spinal pAkt in male mice increased markedly at 30 min, reached a peak at 2 h and returned to baseline at 24 h. The expression of spinal pAkt in female mice increased mildly at 0.5 and 2 h (Fig. 4b), but there was no statistical difference among the 0, 0.5, and 2-h groups, in accordance with the behavior results.

### Cellular localization of spinal-activated Akt

**Fig. 5:**
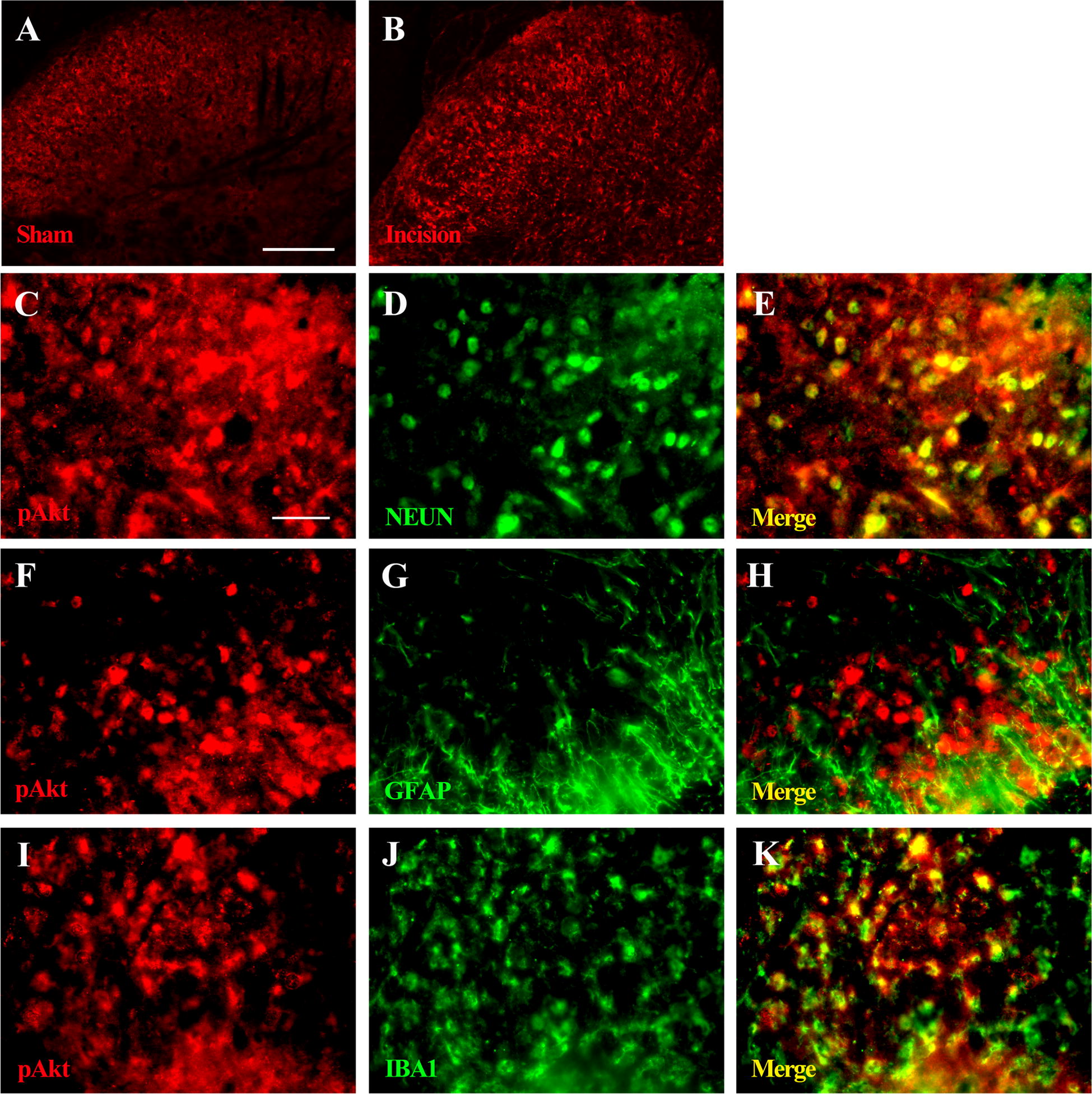
Cellular localization of spinal-activated Akt induced plantar incision in male mice. Double immunofluorescence staining of pAkt (red) was performed with cell-specific markers: neuronal nuclei (NEUN, green) for neurons, ionized calcium-binding adapter molecule 1 (IBA1, green) for microglia, and glial fibrillary acidic protein (GFAP, green) for astrocytes at 2 h after plantar incision in male mice. Immunochemistry with pAkt antibody indicated increased pAkt immunoreactivity levels in the spinal dorsal horn (**B**). Double immunofluorescence staining showed that spinal pAkt was expressed in neurons and microglia (localization with NEUN or IBA1, respectively), but not expressed by astrocytes (no colocalization between pAkt and GFAP). Yellows: colocalization of pAkt with respective cell markers. *n *= 4 mice in each group. Scale bar = 200 μm (**A**, **B**); 50 μm (**C**–**K**)

To identify the cell types that expressed spinal pAkt after plantar incision, double immunofluorescence staining of pAkt was performed with cell-specific markers: neuronal nuclei (NEUN) for neurons, ionized calcium-binding adapter molecule 1 (IBA1) for microglia, and glial fibrillary acidic protein (GFAP) for astrocytes at 2 h after plantar incision. We observed that pAkt induced by plantar incision was primarily expressed in the spinal dorsal horn (Fig. 5A, B, respectively). Furthermore, pAkt was localized with the neuronal marker (NEUN; Fig. 5C–E) and microglia marker (IBA1; Fig. 5I–K), but not with astrocytes (GFAP; Fig. 5F–H).

### Post-surgical inhibition of PI3K attenuated spinal Akt activation induced by plantar incision in male mice

**Fig. 6:**
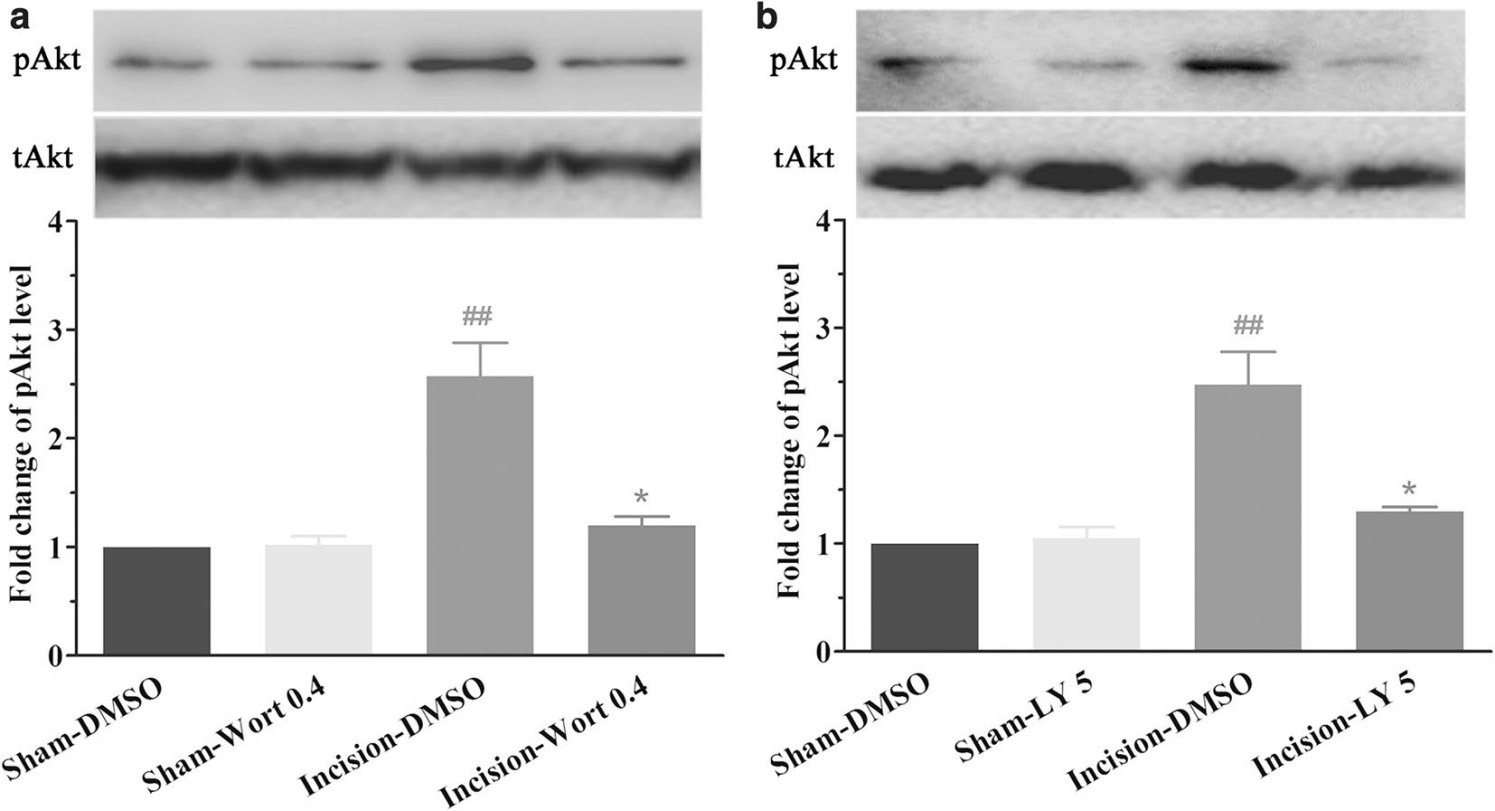
Post-surgical inhibition of PI3K attenuated spinal Akt activation induced by plantar incision in male mice. PI3K inhibitor wortmannin (0.4 μg) or LY294002 (5 μg) or DMSO was intrathecally injected at 45 min after plantar incision. The expression of spinal pAkt was assayed at the 2-h time-point after plantar incision. Post-surgical inhibition of PI3K with both wortmannin (**a**) and LY294002 (**b**) attenuated spinal Akt activation induced by plantar incision. The representative bands (top) and the quantitative data (bottom) for the expression of spinal pAkt after plantar incision. The fold change for the density of each pAkt was normalized to total Akt for each sample. The fold change of pAkt level in Sham-DMSO group was set at 1 for quantifications. ^##^*P *< 0.01 compared with Sham-DMSO group; *P < 0.05, compared with Incision-DMSO group (one-way ANOVA, followed by Bonferroni’s multiple comparison test; *n *= 4 mice in each group). Data are presented as mean ± SEM

Our results showed that the expression of spinal pAkt induced by plantar incision increased at 30 min, reached a peak at 2 h, and returned to baseline at 24 h (Fig. 4) [5]. Therefore, to explore the role of PI3K in the activation of Akt induced by plantar incision, PI3K inhibitor wortmannin (0.4 μg/5 μl), LY294002 (5 μg/5 μl), or DMSO (5%, 5 μl) was intrathecally injected at 45 min after plantar incision in male mice. The expression of spinal pAkt was assayed at the 2 h time-point after plantar incision, using western blot. As compared with Sham-DMSO and Sham-LY group, pAkt was significantly up-regulated in the Incision-DMSO group. Post-surgical treatment with both wortmannin (Fig. 6a) and LY294002 (Fig. 6b) attenuated the up-regulation of pAkt induced by plantar incision. Therefore, PI3K inhibitors attenuated not only the pain behavior induced by plantar incision, but also attenuated the activation of Akt induced by plantar incision.

## Discussion

In this study, we showed that post-surgical inhibition of PI3K could attenuate the pain-related behaviors induced by plantar incision in male mice, which was supported by the following results: (1) post-surgical inhibition of PI3K significantly attenuated mechanical allodynia, thermal hyperalgesia, and cumulative pain scores induced by plantar incision in male mice, and mildly in female mice, and we observed differences in the POP between male and female mice when treated with PI3K inhibitors; (2) post-surgical inhibition of PI3K attenuated the expression of spinal Fos induced by plantar incision in male mice; (3) plantar incision induced a time-dependent activation of Akt in the spinal dorsal horn in male mice, but not in female mice, which was localized with neurons and microglia; (4) post-surgical inhibition of PI3K attenuated spinal Akt activation induced by plantar incision in male mice.

Recent studies have indicated that PI3K is widely expressed in the spinal cord, the most important site of pain modulation [6–14], and the inhibition of spinal PI3K prevented or reversed pain-related behaviors induced by different kinds of stimuli, such as formalin, capsaicin, carrageenan, ephrins, and brain-derived neurotrophic factor (BDNF) [10, 11, 15, 16]. Our previous results showed that plantar incision activated spinal PI3K in laminae I–IV of the spinal cord in a time-dependent manner, and system or spinal blocking of PI3K prevented the primary mechanical and heat hyperalgesia induced by plantar incision [5]. In the present study, post-surgical inhibition of PI3K with wortmannin or LY294002 significantly attenuated the mechanical allodynia, thermal hyperalgesia, and cumulative pain scores induced by plantar incision in male mice (Fig. 1) and mildly in female mice (Fig. 2). The implementation of ERAS protocols promoted opioid-free and multimodal analgesia, in the context of providing adequate pain control [2–4]. Recent studies have demonstrated the critical roles of PI3K in the development and maintenance of pain [17]. PI3K might, therefore, be a novel therapeutic target to reduce POP.

Spinal neuronal sensitization is involved in the development and maintenance of hyperalgesia, and the expression of Fos protein in the spinal dorsal horn is used as a global marker for neuron activation, to examine the effectiveness of analgesic regimens [5, 18, 19]. In our experiment, the number of Fos protein-positive cells increased significantly in the ipsilateral dorsal horn, compared with the sham operation group (Fig. 3a, b), while post-surgical inhibition of PI3K with wortmannin or LY294002 intrathecally, partially attenuated the spinal Fos expression induced by plantar incision (Fig. 3a, b). The vehicle (5% DMSO) and wortmannin or LY294002 did not affect the Fos expression in the sham operation group. These results suggested the analgesic effect of PI3K inhibitors on pain behavior induced by plantar incision.

It has been demonstrated that Akt–a critical downstream signaling molecule of PI3K, is widely expressed in the spinal cord. Our previous results showed that plantar incision significantly increased pAkt expression in the spinal cord, which was prevented by pre-surgical treatment with PI3K inhibitors, wortmannin and LY294002. The present results showed that plantar incision induced a time-dependent activation of Akt in the spinal dorsal horn in male mice (Fig. 4a), but not in female mice (Fig. 4b). The up-regulation of pAkt induced by plantar incision was attenuated by the post-surgical inhibition of PI3K with both wortmannin (Fig. 6a) and LY294002 (Fig. 6b). We, therefore, conclude that PI3K inhibitors attenuated the pain behavior induced by plantar incision in male mice, probably by attenuating the activation of Akt. In the future, we will detect the modulation of spinal Akt on pain behavior induced by plantar incision.

Sex influences on pain and analgesia have been identified in clinical pain conditions, and we observed a similar difference during treatment with the same doses of Wortmannin or LY294002, as the attenuated effects of mechanical allodynia, thermal hyperalgesia, and cumulative pain scores were different in the male and female mice. Even though psychological and social factors certainly play a role in the differences in prevalence and incidence of pain, biological differences likely underlie these observed effects. Female mice use T cells in the spinal cord to mediate pain, whereas male mice preferentially utilize microglia in a similar manner [20]. Our results showed that pAkt induced by plantar incision was primarily expressed in the spinal dorsal horn, and was localized with the neurons (Fig. 5C–E) and microglia (Fig. 5I–K) in male mice, implicating that male mice utilize microglia in the spinal cord to mediate pain. Recognition of the underlying mechanism of sex differences with regard to pain will guide the development of treatments and provide better service for POP. In conclusion, our study provides further evidence for the involvement of PI3K in pain processing and pain modulation, and suggests that PI3K in the spinal dorsal horn may be a novel and potent target for controlling severe POP in male mice, but not in female mice.

## Conclusions

In conclusion, the present study suggests that post-surgical inhibition of PI3K could attenuate the pain-related behaviors induced by plantar incision by suppressing the activation of spinal Akt in male mice. The POP between male and female mice showed a difference based solely on sex when treated with PI3K inhibitors.

## Methods

### Animals

224 male and 120 female Kunming mice (weighing 20–25 g; Guangxi Medical University, Nanning, PR China) were used to establish the mice model of POP according to our previous protocols [5]. The animals were housed with food and water available ad libitum, under a 12-h/12-h light–dark cycle. The experimental and surgical procedures were carried out after approval by the local Animal Care and Use Committee (The People’s Hospital of Guangxi Zhuang Autonomous Region, Nanning; No. 2016-35) and followed the ethical guidelines for the study of experimental pain in conscious animals by the International Association of the Study of Pain. Every effort was made to minimize the suffering of the animals, and the protocol of POP was conducted by briefly anesthetizing with 1.5% to 2% isoflurane (via a nose cone). The samples for immunohistochemistry and western blot were collected after the mice were sacrificed by deeply anesthetizing with sodium pentobarbital (60 mg/kg, intraperitoneal injection, i.p.).

### Establishment of the POP model in mice

As described previously [5, 21], mice were anesthetized with 1.5% to 2% isoflurane delivered via a nose cone. The right hind paw of the mice was disinfected with 10% povidone-iodine solution. A 5-mm longitudinal incision was made through the glabrous skin and the fascia of the plantar foot. Under the fascia, the flexor digitorum brevis muscle was elevated and divided with curved forceps. The skin overlying the muscle was opposed with a single mattress suture. The wound was covered with an antibiotic ointment. Control mice underwent the same procedure as that for the model group, except for the plantar incision.

### Intrathecal (i.t.) drug administration

Wortmannin and LY294002, irreversible and reversible PI3K inhibitors, respectively, were purchased from Sigma (St. Louis). The drugs were dissolved in 5% dimethyl sulfoxide (DMSO). The usage and doses of inhibitors were based on the literature and pilot experiments.

As described by Hylden and Wilcox [22], inhibitors or vehicles was administrated intrathecally into the subarachnoid space between L5 and L6 vertebrae of conscious mice, using a 28-gauge stainless steel needle attached to a 25 μl Hamilton microsyringe. A sudden slight flick of the tail confirmed correct i.t. positioning of the needle tip. A volume of 5 μl DMSO (5%) or drug solution was injected over 30 s into the subarachnoid space, leaving the needle tip in the place for 15 s. Mice with signs of motor dysfunction before the nociceptive test were excluded from the experiments.

### Mechanical allodynia

As we described previously, mice were placed in inverted plastic boxes (5 × 5×8 cm) on a metal mesh floor and habituated for 30 min before behavioral testing. Mechanical allodynia was determined by using calibrated von Frey filaments (0.07-, 0.16-, 0.40-, 0.60-, 1.0-, 1.4-, 2.0-g bending force; Stoelting), starting with a 0.07 g and continuing in ascending order. The investigator was blind to the treatment during behavioral testing. Each filament was applied five times to the plantar aspect of the right paw adjacent to the incision for 1 s with a 10-s interval. A stimulus-related withdrawal of the right paw was considered as a positive response. The paw withdrawal frequency (PWF) to each filament was calculated from five applications. The PWT was considered the force at which PWF ≥ 60%. 2 g was recorded as the PWT if PWF ≤ 60% to all filaments.

### Thermal hyperalgesia

PWL to heat, according to the method described by Hargreaves [23], was used to determine thermal hyperalgesia. In brief, each mouse was placed on a preheated glass platform (28–29.8 °C) within a plastic chamber (7 × 9 × 11 cm). After acclimation for 30 min, a radiant heat source was moved to the middle of the incision area from underneath the glass. The latency required to cause the withdrawal of the hind paw from the heat source was recorded as the PWL. The results of three trials with a 5-min interval provided the average PWL. The heat intensity was adjusted to obtain a baseline between 12 and 18 s (cut off time: 20 s).

### Cumulative pain score (CPS)

The CPS was assessed, as described by Brennan [24]. The CPS was obtained depending on the position in which the right paw was observed during the majority of the 1-min scoring period, repeated every 5 min over 1 h. A score of 2 was given if the foot was completely off the mesh, 0 if the foot was blanched or distorted by the mesh, and 1, if the area of the wound touched the mesh without blanching or distorting. The sum of the 12 scores obtained during the 1 h session was used to assess CPS.

### Immunohistochemistry

For immunohistochemistry, mice were deeply anesthetized with sodium pentobarbital (60 mg/kg, intraperitoneal injection, i.p.) and perfused intracardially with 30 ml saline followed by 100 ml 4% ice-cold paraformaldehyde (PFA) solution in 0.1 M phosphate-buffered saline (PBS). The lumbar L4–5 vertebrae of spinal cord were dissected out, postfixed in the same PFA overnight and transferred into 30% sucrose solution in PBS overnight, at 4 °C. Tissue was cut transversely on a freezing microtome at 20 μm and every fifth section was collected in PBS. Sections were incubated for 30 min in 5% normal goat serum, 0.3% hydrogen peroxide, and 0.3% Triton X-100 in 0.1 M PBS. The sections were, then, incubated in primary polyclone rabbit-anti-Fos antibody (1:1000; Santa Cruz Biotechnology, CA, USA) at 4 °C for 24 h. Sections were washed in PBS for three times and incubated in biotinylated goat anti-rabbit (1:200) at 37 °C for 1 h. Sections were washed again and incubated in an avidin–biotin–peroxidase complex at 37 °C for 2 h. Following washes, sections were incubated in 0.05% diaminobenzidine (DAB) for 5–10 min. The reaction was stopped by washing in PBS. Sections were mounted on gelatin-coated slides, air-dried, dehydrated with alcohol, cleared with xylene, and cover slipped. Every fifth section was picked from a series of consecutive spinal sections, and five sections were counted per spinal cord. The total number of Fos protein in the ipsilateral spinal lamina (I–IV) in each section were counted in all groups. Data were presented as mean ± S.E.M.

For immunofluorescence staining, the sections were incubated with a mixture of either monoclonal anti-phosphorylated Akt antibody (1:200, CST) and anti-neuronal nuclei monoclonal antibody (NEUN; neuronal marker, anti-mouse, 1:200; Chemi-Con), anti- ionized calcium-binding adapter molecule 1 polyclonal antibody (IBA1; microglia marker, anti-goat, 1:250; Abcam), or anti-glial fibrillary acidic protein monoclonal antibody (GFAP; astrocyte marker, anti-mouse, 1:100; CST), at 4 °C for 24 h, followed by a mixture of CY3- and Alexa Fluor 488-conjugated immunoglobulin G (1:200, Abcam) for 2 h at 26 ± 2 °C. Nonspecific staining was determined by excluding the primary antibodies. Sections were rinsed, mounted, and cover slipped with 50% glycerol and stored at − 20 °C in the dark. Images were captured using a fluorescence microscope (BX51, Olympus, Japan).

### Western blot

Mice were deeply anesthetized by sodium pentobarbital (60 mg/kg, i.p.) and decapitated. The spinal cords were hydro-extruded from the vertebral column. The dorsal quadrants of the lumbar spinal cord were dissected and stored in liquid nitrogen. Samples were homogenized in extraction buffer [Tris 20.0 mM; Na_3_VO_4_, 0.03 mM; sucrose, 250.0 mM; MgCl_2_, 2.0 mM; EGTA, 2.0 mM; EDTA, 2.0 mM; phenylmethyl sulfonyl fluoride, 2.0 mM; dithiothreitol, 1.0 mM; and protease inhibitor cocktail, 0.02% (v/v), with pH 7.4]. The homogenized samples were centrifuged at 12,000*g* for 30 min at 4 °C. The supernatants were collected. The protein concentration was determined using BCA protein assay kit (Boster, China), and was dissolved in 4× sample buffer (Boster), and denatured at 95 °C for 5 min. Samples containing an equal amount of total protein (30 μg) were separated by 8% SDS–polyacrylamide gel electrophoresis and transferred onto a polyvinylidene difluoride membrane (PVDF, Millipore, Billerica, MA, USA). All membrane incubations were performed on a rotating plate. After blocking in 5% bovine serum albumin for 1 h at 26 ± 2 °C, the membranes were incubated for 24 h at 4 °C with primary antibody against pAkt (1:1000, CST), or Akt (1:400, Boster). After washing with Tris-buffered saline-Tween (20 nM Tris, 137 nM NaCl, 0.1% Tween), the membranes were incubated in the secondary antibody solution conjugated with horseradish peroxidase (1:5000, Abcam) for 2 h at 26 ± 2 °C. The immune complexes were detected using an ECL Plus kit and exposed to MP-ECL film. Expression of pAkt was normalized to Akt. The blot intensity from the control groups was set as 100%.

### Statistical analysis

Data were expressed as mean ± S.E.M. One-way analysis of variance (ANOVA, followed by Bonferroni`s multiple comparison test) or two-way ANOVA with Bonferroni post-tests were used where appropriate. “Time” was treated as “within-subjects” factor, and “treatment” was treated as a “between-subject” factor. Statistical results were considered significant if *P *< 0.05.

## Data Availability

All data generated or analyzed during this study were included in this article.

## Abbreviations

POP: postoperative pain
PI3K: phosphatidylinositol 3-kinase
Akt: protein kinase B
ERAS: enhanced recovery after surgery
DMSO: dimethyl sulfoxide
PWT: paw withdrawal threshold
PWF: paw withdrawal frequency
PWL: paw withdrawal latencies
CPS: cumulative pain score
NEUN: neuronal nuclei
GFAP: glial fibrillary acidic protein
IBA1: ionized calcium binding adapter molecule 1
BDNF: brain-derived neurotrophic factor
